# The Surgical Significance of Vomiting

**Published:** 1908-05-09

**Authors:** 


### Points in Surgery.

### THE SURGICAL SIGNIFICANCE OF VOMITING.

Stercoraceous or fæcal vomiting is a positive indication of complete occlusion of the intestine, which may be primary, as in acute obstruction, or may supervene on a pre-existing chronic lesion such as a carcinomatous stricture. In either variety of case, as soon as complete occlusion of the bowel occurs, constipation becomes absolute, the appetite is completely lost, and the patient begins to belch and complain of nausea, which persists until vomiting finally occurs. At first the contents of the stomach are evacuated, soon mixed with bile. In the next stage the vomited matter is usually thin and of a brownish colour, or it may resemble pea-soup, or be of a yellow colour like the yolk of an egg. It acquires later what is called an "intestinal odour," and finally it becomes stercoraceous. As Treves pointed out, this does not imply that the vomited matter consists of fæces. Stercoraceous vomiting may occur when the obstruction is quite high up in the jejunum, and is, as a matter of fact, a much more constant symptom in stenosis affecting the small than the large intestine. Stercoraceous vomit is vomit which has an *odour* of unhealthy fæces, which is imparted to it by the results of bacterial decomposition in the obstructed bowel. It is always watery, like the matter voided in diarrhœa, and never contains actual fæcal masses. Lumps resembling scybala are sometimes seen, but are probably masses of coagulated milk stained with bile.

The question of the actual method of production of stercoraceous vomiting has given rise to no little controversy and speculation among medical authors of all times. Galen attributed it to an antiperistaltic action of the bowels (*motus antiperistalticus*). With regard to the question of antiperistalsis, experiments on animals have proved that it can be excited by suitable stimuli. The special stimulus which seems to be demanded is a strong chemical irritant, and a weak solution of skatol is apparently sufficient. Kirstein suggests, therefore, that the stercoraceous vomit contains skatol, which acts in this way. At present, however, the co-operation of antiperistaltic movements in the production of stercoraceous vomiting has not been proved, even if their existence be admitted.

Haguenot of Montpellier (1713) elaborated another theory. His idea is that the intestine above the stenosis is loaded with bowel-contents undergoing bacterial decomposition and liquefied by a copious intestinal secretion. The concomitant nausea starts an attack of vomiting, and the consequent descent of the diaphragm increases the intra-abdominal pressure, so that the accumulated matter must move along the only road that is open to it—*i.e.* towards the stomach. This hypothesis is supported by the fact that cases are not uncommon of intestinal obstruction without stercoraceous vomiting, though an autopsy revealed the small intestine to be full of thin stercoraceous matter. In these cases death occurred before vomiting started at all. There was, therefore, no increase of intra-abdominal pressure, and consequently no regurgitation. But how do the bowel-contents above the stricture become intimately mixed with those higher up? Leichtenstein explains this by his "recoil-contraction" theory (Rückstosscontraction). There is a violent peristalsis of the bowel just above the stricture, which forces the lowest contents against the stricture, and they recoil from the obstacle upwards for a few inches. This is repeated many times, and in the process the intestinal contents are mixed together.

Brinton accepts the theory of Haguenot in the main, but goes one step further by suggesting that the peristaltic and diaphragmatic movements produce two distinct currents in the intestinal canal—a peripheral one travelling downwards and an axial current travelling in the opposite direction.

One need only mention in passing the old theory that stercoraceous vomiting is produced by insufficiency of the ileo-cæcal valve in order to point out that stercoraceous vomit does not contain formed fæces from the large intestine, and that regurgitation of fluid through the ileo-cæcal valve is practically unknown. It can be experimentally shown that if the cæcum is forcibly distended with fluid, the bowel itself will rupture before the valve-flaps give way.

Whatever the method of production of stercoraceous vomiting, one thing is certain and that is that its onset marks the beginning of the end. It is, in fact, the last act of the tragedy which will inevitably conclude with the death of the patient if no operative interference is undertaken. It is true that cases have been recorded where spontaneous recovery has taken place after the occurrence of stercoraceous vomiting, but the probability is that the obstruction in these cases was never of an organic nature. In a typical case the patient, weakened and exhausted by the constant pain and poisoned by the absorption of toxins from his own alimentary canal, gradually sinks until death puts an end to his sufferings.

The following case is a good example of the clinical picture presented. The patient, a man aged sixty-eight, complained of abdominal pain and vomiting of fifteen months' duration. The bowels were only opened once in three weeks and the motions were very constipated. He had passed no blood in his stools, nor had he had diarrhœa. The vomiting had no relation to food. The abdomen was distended and peristalsis of the abdomen was plainly visible, but there was nothing which pointed to the site of the obstruction. The abdomen was opened and an inoperable annular carcinomatous stricture of the transverse colon was found. The abdomen was closed. Ten days after the operation the occlusion of the intestine appeared to become absolute, and the vomit soon acquired a stercoraceous odour. The patient became rapidly thinner and weaker. Three days later his temperature rose suddenly to 107.6° F., and he became comatose. He died the same evening.

